# Fiber diameters and parallel patterns: proliferation and osteogenesis of stem cells

**DOI:** 10.1093/rb/rbad001

**Published:** 2023-01-12

**Authors:** Zhanghong Gu, Suna Fan, Subhas C Kundu, Xiang Yao, Yaopeng Zhang

**Affiliations:** State Key Laboratory for Modification of Chemical Fibers and Polymer Materials, Shanghai Engineering Research Center of Nano-Biomaterials and Regenerative Medicine, College of Materials Science and Engineering, Donghua University, Shanghai 201620, People’s Republic of China; State Key Laboratory for Modification of Chemical Fibers and Polymer Materials, Shanghai Engineering Research Center of Nano-Biomaterials and Regenerative Medicine, College of Materials Science and Engineering, Donghua University, Shanghai 201620, People’s Republic of China; I3Bs-Research Institute on Biomaterials, Biodegradables and Biomimetics, Headquarters of the European Institute of Excellence on Tissue Engineering and Regenerative Medicine, University of Minho, Barco, Guimarães 4805-017, Portugal; State Key Laboratory for Modification of Chemical Fibers and Polymer Materials, Shanghai Engineering Research Center of Nano-Biomaterials and Regenerative Medicine, College of Materials Science and Engineering, Donghua University, Shanghai 201620, People’s Republic of China; State Key Laboratory for Modification of Chemical Fibers and Polymer Materials, Shanghai Engineering Research Center of Nano-Biomaterials and Regenerative Medicine, College of Materials Science and Engineering, Donghua University, Shanghai 201620, People’s Republic of China

**Keywords:** single-layer and parallel arranged fiber patterns, non-fouling background, cell–fibrous material interaction, cell differentiation

## Abstract

Due to the innate extracellular matrix mimicking features, fibrous materials exhibited great application potential in biomedicine. In developing excellent fibrous biomaterial, it is essential to reveal the corresponding inherent fiber features’ effects on cell behaviors. Due to the inevitable ‘interference’ cell adhesions to the background or between adjacent fibers, it is difficult to precisely reveal the inherent fiber diameter effect on cell behaviors by using a traditional fiber mat. A single-layer and parallel-arranged polycaprolactone fiber pattern platform with an excellent non-fouling background is designed and constructed herein. In this unique material platform, the ‘interference’ cell adhesions through interspace between fibers to the environment could be effectively ruled out by the non-fouling background. The ‘interference’ cell adhesions between adjacent fibers could also be excluded from the sparsely arranged (SA) fiber patterns. The influence of fiber diameter on stem cell behaviors is precisely and comprehensively investigated based on eliminating the undesired ‘interference’ cell adhesions in a controllable way. On the SA fiber patterns, small diameter fiber (SA-D1, D1 means 1 μm in diameter) may seriously restrict cell proliferation and osteogenesis when compared to the middle (SA-D8) and large (SA-D56) ones and SA-D8 shows the optimal osteogenesis enhancement effect. At the same time, the cells present similar proliferation ability and even the highest osteogenic ability on the densely arranged (DA) fiber patterns with small diameter fiber (DA-D1) when compared to the middle (DA-D8) and large (DA-D56) ones. The ‘interference’ cell adhesion between adjacent fibers under dense fiber arrangement may be the main reason for inducing these different cell behavior trends along with fiber diameters. Related results and comparisons have illustrated the effects of fiber diameter on stem cell behaviors more precisely and objectively, thus providing valuable reference and guidance for developing effective fibrous biomaterials.

## Graphical abstract

**Figure:**
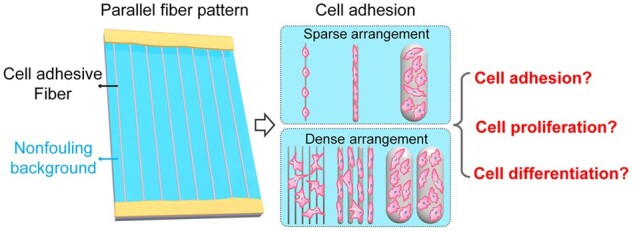

## Introduction

Many essential components of the extracellular matrix (ECM), such as collagen, elastin and keratin, present fibrous structures, which could provide necessary biological and physical support for cells [1–5]. Cells could constantly respond to varied ECM features to change corresponding gene expression and cell function, thus finally achieving desired tissue function and homeostasis maintenance [6–8]. Just because of the inherent ECM-mimicking structure, fibrous material has been studied and utilized as an essential biomaterial [9–15] and has been widely used in biomedical fields such as surgical sutures [16–18], wound dressings [19–25] and tissue engineering [5, 26–31]. The diameter of the widely used fibrous biomaterials usually ranges from several hundred nanometers to several microns [32–34], and the diameter of the surgical suture can reach tens of microns [17].

The primary study of cell–material interactions, such as precisely revealing varied material cues’ effect on cell behaviors could provide valuable reference and novel strategies for the effective design and fabrication of biomaterials [35–38]. As for developing excellent fibrous biomaterials, it is also essential to accurately reveal the inherent fiber features’ effects on cell behaviors, such as the fiber diameter and arrangement. Until now, many efforts have been made in this field. For example, Li *et al.* [39] have prepared highly oriented or randomly arranged fiber mats with micro- (1.3–2.4 μm) or nano (0.3–0.4 μm) diameters by using electrospinning and found that the oriented fibers could significantly induce endothelial cells alignment along with corresponding fibers. Moreover, compared to the randomly arranged fiber mat, the oriented fibers could also enhance cell proliferation for both the micro and nano-sized fiber mats. By using randomly placed electrospinning fiber mats with varying fiber diameters (283–1492 nm), Christopherson *et al.* [40] investigated the ‘fiber diameter’ effect on cell neurogenic differentiations. Results showed that the neural stem cells could be more feasibly differentiate into oligodendrocytes on the fiber mat with 283 nm in fiber diameter (relatively small) and more easily differentiate into neuronal cells on that with relative larger fiber diameters (749 and 1492 nm). In addition, a higher degree of cell spreading was also observed on the fiber mats with small diameters (283 nm) compared to larger ones [40]. With the help of electrospinning techniques, some other researchers have also reported that the ‘fiber diameter’, ‘aligned orientation’ and ‘random orientation’ feature in traditional fiber mat platforms could have a profound effect on cell behaviors [41–44].

However, it needs to be noted that there were inevitable ‘interference’ cell adhesions in those existing studies for precisely revealing the inherent fiber diameter and other features’ effect on cell behaviors, such as the ‘interference’ cell adhesions through interspace between fibers to the background (tissue culture plates as an example), and the ‘interference’ cell adhesions between adjacent fibers (both in the left and right positions or up and down places), as schematically illustrated in Supplementary Fig. S1. These ‘interference’ adhesions could probably disrupt the conclusion of fiber diameter effect on cell behaviors, making it challenging to reflect related cell–material interactions accurately. Due to the lack of an effective material platform, few reports have studied the inherent fiber diameter effect on cell behaviors based on excluding these undesired ‘interference’ cell adhesions.

**Figure 1.**
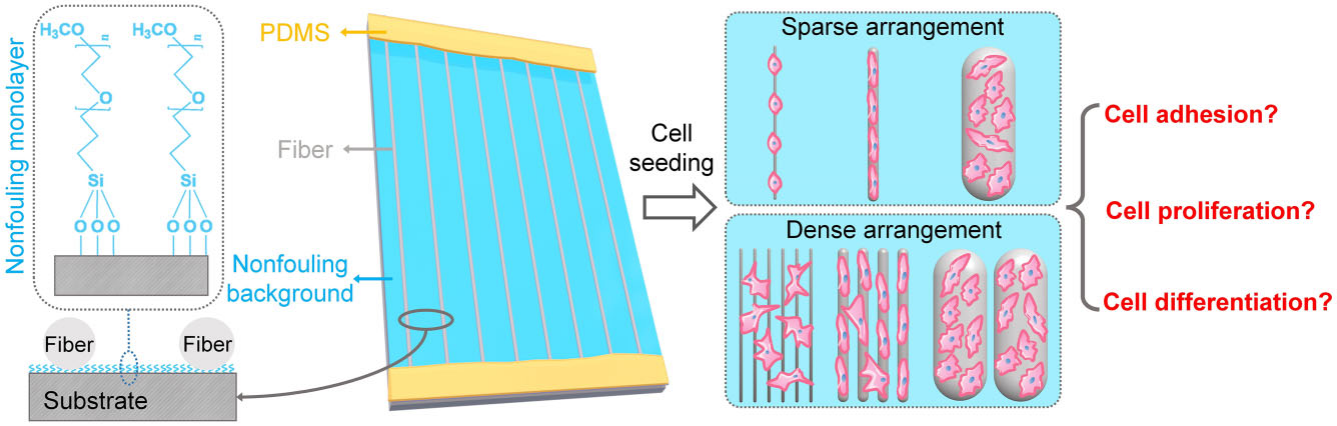
Schematic illustration of the ideas to precisely and comprehensively explore the effects of fiber diameter on stem cell behaviors under a sparse or dense arrangement using the single-layer and parallel-arranged fiber patterns with a non-fouling background.

A single-layer and parallel-arranged fiber pattern platform with a non-fouling background was first designed and constructed in this report to precisely reveal the inherent fiber diameter effect on cell behaviors. This could rule out the above-mentioned ‘interference’ cell adhesions in a controllable way, as schematically illustrated in Fig. 1. Specifically, a modified substrate (background) with excellent non-fouling properties was obtained by grafting oligo-ethylene glycol on the glass surface. Combined with the electrospinning, dry spinning and wet spinning technologies of polycaprolactone (PCL, one kind of popularly used biomaterials), single-layer and parallel arranged PCL fiber patterns with typical fiber diameters (1–56 μm) according to the ranges of usually used fibrous biomaterials were fabricated on the modified substrate. Both sparsely arranged (SA, with average interspacing distance between adjacent fibers significantly larger than 120 μm) and densely arranged (DA, with average interspacing space between adjacent fibers obviously smaller than 50 μm) fiber patterns were fabricated for each fiber diameter. By further using polydimethylsiloxane (PDMS), two ends of the fibers were tightly fixed on the edges of the modified substrate, thus achieving stable single-layer and parallel-arranged fiber patterns with an excellent non-fouling background. In this unique material platform, the ‘interference’ cell adhesions through interspace between fibers to the environment could be effectively ruled out by the non-fouling background. And the interference cell adhesions between adjacent fibers could be excluded under the condition of sparse fiber arrangement. Therefore, the influence of inherent fiber diameter on cell behaviors could be precisely investigated based on eliminating the undesired ‘interference’ cell adhesions on the SA fiber patterns. The impact of fiber diameter combined with cross-adhesion between adjacent fibers on cell behaviors could also be comprehensively studied on the DA fiber patterns, which could be used to mimic traditional fiber mat platforms with partial ‘interference’ cell adhesions to some extent, as schematically shown in Fig. 1. Then, rat bone marrow mesenchymal stem cells (bMSCs) will be chosen as model cells to precisely and comprehensively investigate the fiber diameter effects on stem cell adhesion, proliferation and osteogenesis under sparse or dense fiber arrangement.

## Experiments and methods

### Materials

PCL (Mn ≈ 80 000 Da), FITC-phalloidin, 2-(4-amidinophenyl)-6-indolecarbamidine dihydrochloride (DAPI), paraformaldehyde and Triton X-100 from Sigma (USA), glass slides (26 mm × 23 mm) from Jiangsu Feizhou Glass Plastic Co., LTD (China), toluene, sulfuric acid, 30% hydrogen peroxide, triethylamine, dichloromethane (DCM), *N,N*-dimethylformamide (DMF) and ethanol from Sinopharm Chemical Reagent Co., LTD (China), 2-[methoxy(polyethyleneoxy)_6–9_ propyl]trimethoxysilane (MPTES) from Gelest (USA), the Sylgard 184 poly(dimethylsiloxane) (PDMS) and curing agent from Dow Corning (USA), fetal bovine serum (FBS), minimum essential medium *α* (*α*-MEM), Dulbecco’s modified Eagle’s medium (DMEM), penicillin, streptomycin 0.25% trypsin–EDTA and phosphate-buffered saline (PBS) from Gibco (USA), the MagneSil^@^ Total RNA mini isolation system from Promega (USA) and primary rat bMSCs from Shanghai AllCells Biotech Co., Ltd (China) were purchased for this experimentation.

### Fabrication of the non-fouling substrate

The non-fouling substrate was obtained by grafting MPTES on the surface of glass slides. Firstly, glass slides (26 mm × 22 mm) were cleaned with piranha solution (hydrogen peroxide:concentrated sulfuric acid = 3:7) for about 30 min, then ultrasonically washed with deionized water (10 min) and dried with N_2_ gas. Afterwards, the slides were treated with oxygen plasma (15 min, 100 W) and quickly immersed in Milli-Q water to generate hydroxyl groups on the glass surface. The substrate was then placed in a reaction vessel containing the corresponding reaction liquid in an N_2_ atmosphere, composed of 10 mM MPTES in anhydrous toluene and 1% of triethylamine as a catalyst. The reaction vessel was then incubated in a water bath for at least 48 h (about 60°C). After that, the glass substrate was ultrasonically bathed in toluene and ethanol solution separately (each for about 5 min), to remove the non-covalently adsorbed MPTES. Then, the non-fouling glass substrates were finally obtained after drying with N_2_ gas.

### Preparation of the PCL solution

The PCL solution for electrospinning and dry spinning was prepared by dissolving PCL in a mixed solvent of DCM and DMF (75/25, v/v) at 18 wt% concentration. The PCL solution for wet spinning was prepared by dissolving PCL in DMF at 6 wt% concentration.

### Construction of single-layer and parallel-arranged fiber patterns with non-fouling background

In fibrous biomaterials, electrospinning fiber scaffolds (about several hundred nanometers to several microns in fiber diameter) have been popularly used in wound dressings and tissue engineering [25, 26, 31, 45]. And the widely used surgical sutures usually present tens of microns in fiber diameter [15, 16, 18]. In addition, it has been reported that single microstripes with about 10 μm in width showed the optimal cell contact guidance effect and migration behaviors in the investigated range of 4–100 μm [46]. Based on these common range of fibrous biomaterials (several hundred nanometers to tens of microns) and cell adhesion behaviors on the single microstripes with different widths, single-layer and parallel-arranged fiber patterns with typical fiber diameters of about 1 μm (D1), 8 μm (D8) and 56 μm (D56) were fabricated on the non-fouling glass substrate by the combination of electrospinning, dry spinning and wet spinning technologies (schematically illustrated in Supplementary Fig. S2). In addition, the SA (interspacing distances between adjacent fibers were more significant than 120 μm) and DA (interspacing spaces between adjacent fibers were relatively more minor than 50 μm) fiber patterns were designed and fabricated for each diameter group.

The fiber patterns with a small fiber diameter of about 1 μm (SA-D1 and DA-D1) were prepared via electrospinning by introducing a gap (250 mm in width) formed by two parallel arranged metal plates at the conductive collector according to the previous report [47] (schematically presented in Supplementary Fig. S2a). A flat-tipped needle (0.29 mm in inner diameter) was placed upward at 130 mm away from the grounded parallel plate and connected to a power supply (12 kV). A 5-ml syringe attached to the needle mentioned above was equipped with an injection pump (KDS210P, KD Scientific, USA). The prepared PCL solution for electrospinning was extruded through the needle at a constant rate of 0.6 ml/h. To obtain SA fiber patterns, uniaxially oriented fibers were collected on the non-fouling substrate between the two parallel-arranged metal plates for about 10 s. For the DA fiber pattern fabrication, the corresponding collection time would be extended to about 60 s.

The fiber patterns with a middle fiber diameter of about 8 μm (SA-D8 and DA-D8) were prepared by dry spinning. Firstly, the prepared PCL solution for dry spinning was added into a 5-ml syringe and extruded from the metal needle (0.25 mm in inner diameter) at a constant rate of 0.04 ml/h by an injection pump (KDS210P, KD Scientific). Then the monofilament was drawn out and reeled at 12 cm/s on the collection roller with the non-fouling substrate fixed. To effectively regulate the interspacing between collected fibers on the substrate, a screw slide platform set with a guide hook was loaded between the needle and the collection roller (Supplementary Fig. S2b). The distance between collected fibers can be effectively controlled by adjusting the moving speed of the screw. In this study, the SA fibers on the substrate were prepared by setting the screw moving speed at about 1.4 cm/min. The DA fibers were fabricated by reducing the corresponding rate to about 0.2 cm/min.

The fiber patterns with large fiber diameters of about 56 μm (SA-D56 and DA-D56) were prepared by wet spinning. The prepared PCL solution for wet spinning was added into a 5-ml syringe and extruded from a metal needle (0.15 mm in inner diameter) into the water coagulation bath at a constant rate of about 1.2 ml/h an injection pump (KDS210P, KD Scientific). The wet-spun fibers were reeled at a speed of about 2 cm/s and then the fibers were stretched 3-fold in air and collected on the collection roller fixed with the non-fouling substrate. A screw slide platform was also loaded between the drawing roller and the collection roller to effectively regulate the spacing of collected fibers by adjusting the moving speed of the screw (Supplementary Fig. S2c). The SA fibers on the substrate were prepared by setting the screw moving speed at about 1.0 cm/min and the DA fibers were fabricated by reducing the corresponding rate to about 0.1 cm/min.

After corresponding fiber collection on the non-fouling substrate, PDMS was used to tightly fix the ends of fibers onto the edges of the non-fouling substrate. Specifically, after a suitable amount of PDMS prepolymer (Sylgard 184 poly(dimethylsiloxane):curing agent = 10:1) coating on the edge regions (also with fiber ends on there) of the substrate, it was placed in a 50°C oven for about 1 h to complete the curing process of PDMS prepolymer and finally obtained stable single-layer and parallel arranged fiber patterns with excellent non-fouling background.

### Characterization of the fiber patterns

The diameter, morphology and arrangement features of the fibers in fiber patterns were observed by SEM (FlexSEM 1000II, Hitachi, Japan). After being dried in the air, the samples were sputtered with platinum at the current of 10 mA for the 60 s and observed using SEM at a voltage of 5 kV.

The wide-angle X-ray diffractograms were obtained on a D8 diffractometer (Bruker, USA) with Cu Ka radiation in the 2*θ* range of 5–50° at 40 kV to characterize the crystallinity of fibers with different diameters.

### Cell culturing

The bMSCs were cultured in *α*-MEM medium with 10% FBS, 1% penicillin and 1% streptomycin at 37°C with a 5% CO_2_ atmosphere in a humidified incubator. Cells were digested with 0.25% trypsin–EDTA and passaged at nearly 80% confluence. Only passages 2–4 of primary bMSCs were used in the later experiments.

### Cell seeding and culturing on the fiber patterns

The fiber patterns were placed in a six-well plate (one design per well) and fixed to the bottom using custom polytetrafluoroethylene rings. The fixed fiber patterns were sterilized with ethanol solution (75% in Milli-Q water) for about 30 min and then washed thoroughly with PBS. Then, cells were seeded into each well with a density of 5 × 10^4^ cells/ml (a total 2 ml per well). After the cells were cultured in the incubator for about 4 h, the suspended cells and corresponding culture medium were removed. Then the same amount of fresh cell culture medium was added into the well for further cell culture. During subsequent cell culture, the culture medium was changed every 2 days.

### Cell behaviors evaluation by fluorescence micrographs

#### Cell staining and photography

To evaluate related cell adhesion and proliferation on the constructed fiber patterns, 12 and 60 h of cell culture have been chosen as two specific evaluation time points according to previous literature [12, 41, 48]. Specifically, after 12 h or 60 h of culture, indicated samples were fixed with 4% paraformaldehyde for 15 min at room temperature. Then, the cells were permeabilized with 0.1% v/v Triton X-100 in PBS for 5 min before staining. Filamentous actin (F-actin) was stained with FTIC-phalloidin (1 μg/ml) for 30 min. Then cell nuclei were stained with DAPI (4 μg/ml) for 5 min to evaluate corresponding cell adhesion and cell number on each fiber pattern. The fluorescence micrographs of the stained samples were observed by a fluorescence microscope (DMi8, Leica, Germany).

#### Cell aspect ratio and orientation evaluation

In the fluorescence micrographs of F-actin, the boundaries between cells are difficult to distinguish due to the relatively high cell density on the fibers. Thus, according to previous reports, the aspect ratio and orientation of cell nuclei were measured to reflect the corresponding cell aspect ratio and orientation characteristics [46, 49]. The aspect ratio and orientation angles (*θ*) of cells on different fiber patterns were measured by the software Image J based on the captured fluorescence micrographs of cell nuclei. The orientation angle of the underneath fiber was defined as 0°. In addition, the order parameter (*S*) was calculated for evaluating the orientation degree of cell nuclei on the indicated fiber patterns with the formula *S *=* *2〈cos^2^*θ*〉 – 1 [46], where *θ* is the orientation angle of a cell nucleus. In principle, *S* is 0 in the case of random orientation and 1 when cell nuclei are completely oriented along the underneath fiber.

#### Cell proliferation evaluation

Based on the fluorescence micrographs of cells cultured on the indicated fiber patterns for 12 h or 60 h, the cell number was measured by Image J software according to previous reports [50, 51]. Then, the corresponding cell density (*D*) was calculated using the total cell number divided by the fiber surface area available for cell adhesion. Herein, the indicated fiber surface area is calculated according to the number of fibers multiplied by the surface area of a single fiber. Cell proliferation rate (*P*) was obtained referring to the similar idea of this report [48]. It was finally calculated through the changing of cell density between 12 h (*t*_1_) and 60 h (*t*_2_) according to the following formula:

$$P=\frac{D\left(t_{2}\right)-D\left(t_{1}\right)}{D\left(t_{1}\right)}\times 100\%\;\left(t_{2}>t_{1}\right)$$

where *P* is the cell proliferation rate, and *D* is the cell density on the indicated fiber patterns at the initial (*t*_1_) or end (*t*_2_) state.

### Cell adhesion evaluation by SEM micrographs

The adhesion and morphology of cells cultured on fiber patterns were also carefully observed by SEM (FlexSEM 1000II, Hitachi, Japan). Briefly, bMSCs seeded on the fiber patterns for 12 h were gently rinsed three times with PBS and fixed with 2.5 vol% paraformaldehyde in PBS at 4°C for 2 h, and then dehydrated with a series of ethanol solutions (30, 50, 70, 75, 80, 90 and 100 vol% in water) successively, each for 10 min. After being dried in the air, the samples were sputtered with platinum at the current of 10 mA for 60 s and then observed using SEM at a voltage of 5 kV.

### Gene expression evaluation

To further evaluate the fiber diameter effect on cell osteogenesis, reverse transcriptase polymerase chain reaction (RT-PCR) was applied to measure the expression of osteogenic-related genes, such as ALP, Col I, OPN and Runx2. Briefly, the total RNA of indicated samples were isolated by MagneSil^@^ Total RNA mini-isolation system through recommended procedures [52–54] after 4 days of cell culture. Following cDNA synthesis and quantitative fluorescence testing by Shanghai Daixuan Biotechnology Co., Ltd, data were analyzed using the 2^−ΔΔCt^ method, and *β*-actin was chosen as the housekeeping gene [55, 56]. The primers of the osteogenic characteristic and housekeeping genes used in this study are listed in Table 1.

**Table 1.** RT-PCR primer sequences of the tested genes

	Forward primer (5′–3′)	Reverse primer (5′–3′)
ALP	GAAAGAGAAAGACCCCAGTTAC	ATACCATCTCCCAGGAACAT
Col I	TTAACAAGGGAGGAGAGAGTG	GGAGGGTTTCAGAAGAGAGA
OPN	CGCATTACAGCAAACACTCAG	GTCATCGTCGTCGTCATCAT
Runx2	CGAAATGCCTCTGCTGTTAT	CGTTATGGTCAAAGTGAAACTCT
*β*-Actin	CCTCTATGCCAACACAGT	AGCCACCAATCCACACAG

### Statistical analysis

All statistical data were presented by mean value ± standard deviation and *n *=* *3 for each group unless otherwise indicated. One-way ANOVA analysis was applied to evaluate the differences between indicated groups. A difference was regarded as significant when *P *<* *0.05, as usual.

## Results and discussion

### Fabrication of single-layer and parallel-arranged fiber patterns with excellent non-fouling background

To precisely and comprehensively investigate the effects of fiber diameter on stem cell behaviors, a non-fouling background for this fiber pattern platform was designed to effectively exclude the ‘interference’ cell adhesions through interspace among fibers to the background (such as tissue culture plate). In addition, single-layer and parallel-arrangement features were designed to eliminate the ‘interference’ cell adhesions between adjacent fibers on the up and down positions. When both objectives mentioned above were achieved, the only possible ‘interference’ cell adhesion is the adhesion between adjacent fibers on the left and right positions. On traditional pattern surfaces with cell–adhesion-contrast properties for revealing cell–material interactions, it has been reported that the interspacing between isolated islands smaller than 50–70 μm could easily induce cell adhesions to cross adjacent patterns. In comparison, more than 100–120 μm could effectively avoid those cross-adhesions [35, 48, 52]. Based on this knowledge, we designed two fiber patterns with single-layer and parallel fiber arrangements. One kind is SA fiber patterns with an average interspacing distance between adjacent fibers significantly more prominent than 120 μm, which could be used to guarantee no ‘interference’ cell adhesion happens. The other kind is DA fiber patterns with an average interspacing distance between adjacent fibers smaller than 50 μm, which could be used to mimic traditional fiber mat platforms with partial ‘interference’ cell adhesion.

In order to realize the preparation of fibers with large diameter ranges (from 1 μm to about 56 μm), the electrospinning, dry spinning and wet spinning of PCL were adopted. As for the electrospinning by introducing a gap formed by two parallel arranged metal plates at the conductive collector, the regulation of interspacing between adjacent fibers can be realized by adjusting the deposition time of fibers, as shown in Supplementary Fig. S2a. As for the dry spinning and wet spinning, a screw slide platform fixed with a guide hook was loaded between the needle and the collection roller. The distance between adjacent fibers can be controlled by adjusting the moving speed of the screw (Supplementary Fig. S2b and c).

**Figure 2.**
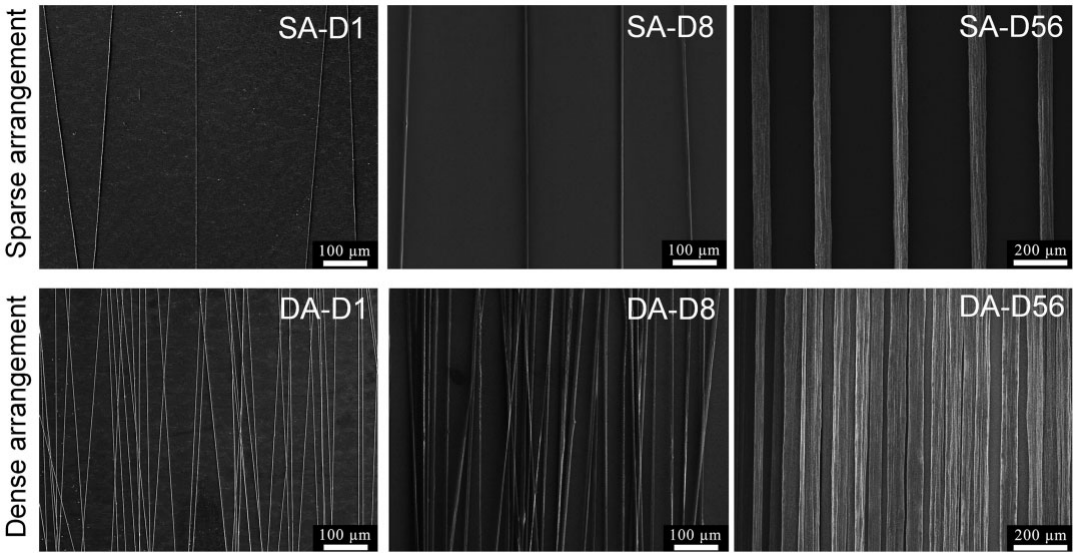
SEM micrographs of the fabricated single-layer and parallel-arranged fiber patterns with varying diameters. SA-D1, SA-D8 and SA-D56: sparsely arranged fiber patterns with about 1, 8 and 56 μm in fiber diameter, respectively; DA-D1, DA-D8 and DA-D56: densely arranged fiber patterns with about 1, 8 and 56 μm in fiber diameter, respectively.

SEM of the fabricated single-layer and parallel-arranged fiber patterns with typical diameters were illustrated in Fig. 2. The fiber diameter and interspacing distance between adjacent fibers were measured and analyzed using SEM micrographs. Statistical results demonstrated that the average fiber diameters of D1 (SA-D1 and DA-D1), D8 (SA-D8 and DA-D8) and D56 (SA-D56 and DA-D56) were about 1, 8 and 56 μm, respectively (see Supplementary Table S1). The average interspacing distances between adjacent fibers in the SA fiber pattern (SA-D1, SA-D8 and SA-D56) were about 145–166 μm (Supplementary Table S1). Thus could effectively avoid corresponding cross-adhesions between adjacent fibers theoretically. In contrast, DA fiber patterns (DA-D1, DA-D8 and DA-D56) were about 8–19 μm (Supplementary Table S1). This could quickly induce cell adhesions to cross adjacent fibers theoretically. Therefore, the fiber diameters and interspacing distances between adjacent fibers could meet the academic and pre-designing requirements.

**Figure 3.**
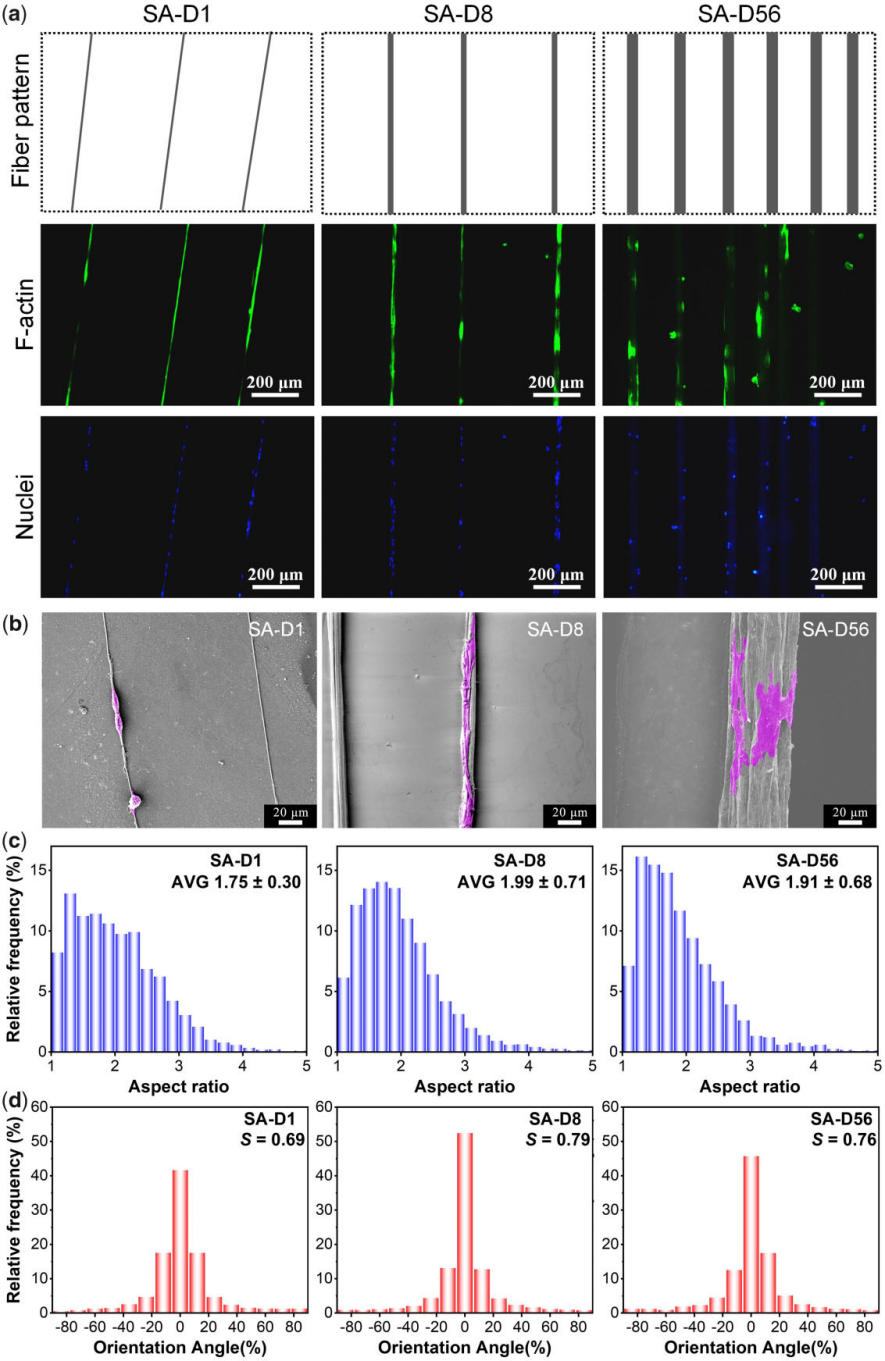
Adhesion of bMSCs on the SA fiber patterns with different fiber diameters after 12 h of culture. (**a**) Fluorescence micrographs of the cells and corresponding cartoon presentation of the fiber patterns. (**b**) SEM micrographs. (**c**) Statistical results of the aspect ratio distribution of cells. The data of the histograms came from the nuclear aspect ratio of bMSCs. Data on the top-right corner represents mean ± SD of the aspect ratio. (**d**) Statistical results of the orientation angle distribution of cells. The data of the histograms came from the nuclear orientation of bMSCs, and the zero angles were defined along the fiber direction; *S* refers to the order parameter of cell orientation.

In addition, we also carefully observed the surface morphology features of the fabricated fibers. SEM micrographs of the surface of the single fiber in the fabricated fiber patterns are illustrated in Supplementary Fig. S3. The small (D1) and middle (D8) diameter fibers presented a similarly smooth surface. As for the large diameter fiber (D56, fabricated by wet spinning), the fibers were formed in a water coagulation bath, accompanied by the slow diffusion of the solvent into the coagulation bath. Probably due to the coagulation bath and specific stretching effects, the surface of D56 fiber showed some shallow and irregular groove-like structures. The detailed morphology information showed that the groove-like structure's depth is relatively shallow and irregular (see Supplementary Fig. S3). Unlike the deep and regular groove structures with solid cell contact guidance effects reported before [57, 58], this kind of groove-like structure on D56 fibers might only slightly affect corresponding cell contact guidance. In addition, the tested order parameter of cell orientation on D56 fibers is smaller than that of the D8 fibers (see Figs 3d and 6d), which also indirectly indicated that the shallow and irregular structures on D56 fibers might only slightly affect related contact guidance. An X-ray diffractometer was also used to characterize the crystallinity of the fabricated PCL fibers. Statistical results showed that there was no significant difference between the crystallinity of D1, D8 and D56 fibers (see Supplementary Fig. S4). Comprehensively, these kinds of fiber patterns could be used to effectively reveal the fiber diameter effects on cell behaviors when excluding other possible ‘interference’ material or cell adhesion cues as far as possible.

### Effects of fiber diameter on stem cell adhesion and proliferation under sparse arrangement

After 12 h of culture on the SA fiber patterns with varied diameters, cell cytoskeleton (F-actin) and nuclei were stained. Typical fluorescence micrographs and SEM micrographs are shown in Fig. 3a and b. Results indicated that cells could only adhere to the indicated fibers and no crossing adhesion between adjacent fibers happened. Even after 60 h of culture, a similar cell adhesion phenomenon could still be maintained (Supplementary Fig. S5). So, the SA fiber patterns fabricated here could effectively exclude the ‘interference’ cell adhesions to the background or between adjacent fibers, thus providing a powerful material platform to reveal the fiber diameter cues’ effect on cell behaviors.

In the fluorescence micrographs of F-actin, the boundaries between cells are difficult to distinguish due to the relatively high cell density on the fibers. According to previous reports, cell nuclei's aspect ratio and orientation were measured to indirectly reflect the corresponding cell aspect ratio and orientation characteristics [46, 49]. Statistical results of the aspect ratio and the orientation angle distribution of cell nuclei are shown in Fig. 3c and d. The average aspect ratios were about 1.75, 1.99 and 1.91 for the cells on the SA-D1, SA-D8 and SA-D56, respectively. In addition, the order parameters (*S*, orientation degree along corresponding fibers) were about 0.69, 0.79 and 0.76 for the cells on the SA-D1, SA-D8 and SA-D56, respectively. These results indirectly indicated that most of the cells could align with corresponding fibers (SA-D1, SA-D8 and SA-D56), and SA-D8 showed the optimal cell contact guidance effect, as it showed the maximum aspect ratio and order parameter of cell orientation.

Combined with the SEM micrographs (see Fig. 3b), it could be found that cells on the single tiny diameter fibers (SA-D1) were restricted seriously and contracted into a bead-like morphology, which is probably why the cells on these fibers presented the most minor aspect ratio and order parameter. Cells on the single middle diameter fibers (SA-D8) showed apparent elongated morphology, probably because the cell adhesion restriction and contact guidance could obtain suitable equilibrium on these fibers, just similar to the cell adhesion on the appropriate microstripes [46]. Finally, inducing the most significant aspect ratio and order parameter of cells on the SA-D8. Cells on the single large diameter fibers (SA-D56) presented relative ‘free’ spreading, while the long axes of most cells still orientated along with the fibers, probably because of the contact guidance effect of the fibers, thus showing the medium value of aspect ratio and orientation order parameter when compared to that of the SA-D1 and SA-D8.

**Figure 4.**
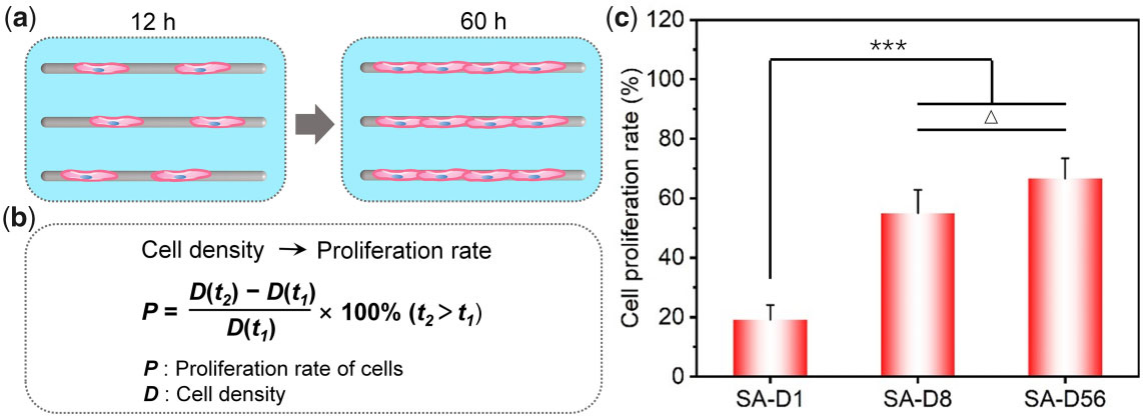
Proliferation of bMSCs on the SA fiber patterns after 60 h of culture. (**a**) Cartoon presentation to illustrate the proliferation of cells. (**b**) Schematic presentation of the calculation of cell proliferation rate from the change of cell density (*D*, cell number divided by the total adhesive area of corresponding fibers). (**c**) Statistical results of the cell proliferation rate. ‘***’: *P *<* *0.001, ‘Δ’: *P *>* *0.05.

After 60 h of culture, the cell proliferation rate was calculated via cell density changing from 12 to 60 h, as schematically shown in Fig. 4a and b. As the fiber diameter increased, the cell proliferation rates were 19.2 ± 4.9%, 55.0 ± 7.9% and 66.7 ± 6.8%, respectively. In brief, the proliferation rate of cells on the small diameter fibers (SA-D1) was significantly lower than that on the middle (SA-D8) and large-diameter fibers (SA-D56). And there was no significant difference in cell proliferation between the statistical results of SA-D8 and SA-D56, while the average cell proliferation rate on the SA-D56 was a little higher (see Fig. 4c).

According to previous reports, immunofluorescence staining of vinculin showed that the size of the mature focal adhesion spot was about a few microns [59, 60]. Therefore, we speculated that although cells could sense and anchor to the single fine fibers in SA-D1, the amount of mature focal adhesion could seriously be decreased own to the limited adherent regions. And the minimal cell adhesions would affect the signal transduction involved in integrin, which could further prompt bead-like cell morphology and reduce cell stress, so the cells also presented poor proliferation ability (see Fig. 4c). With the increase in fiber diameter, the SA-D8 and SA-D56 fiber patterns provided more areas for cell adhesion and growth, resulting in a higher cell proliferation rate. In addition, due to the significant increase of the fiber surface (cell adhesion region) on SA-D56, the cell density and cell–cell contact decreased significantly. It was reported that larger cell spreading areas and more cell–cell communication favorable to cell proliferation [35, 48]. As a result, compared to SA-D8, cells on the SA-D56 fibers presented more extensive spreading while having lower cell–cell contact. The comprehensive effects of these cues showed the cells a similar proliferation rate during the tested period between these two fiber patterns, while the average value on SA-D56 was a little higher (see Fig. 4c).

### Effects of fiber diameter on stem cell osteogenesis under sparse arrangement

To evaluate the effects of fiber diameter on stem cell osteogenesis more intuitively, bMSCs were cultured on the indicated fiber patterns with varying fiber diameters in a growth medium [52] for about 4 days. Then, the expression of osteogenic specific genes (ALP, Col I, OPN, Runx2) of cells was detected by RT-PCR technology. ALP is a particular protein in the early stage of osteogenic differentiation, which mainly promotes cell maturation and calcification. Col I is the primary collagen in the osteogenesis stage and the most crucial collagen component in the bone matrix. OPN is a bone matrix salivary protein that also plays a vital role in biomineralization, and Runx2 is also essential in the regulation of bone formation and bone remodeling [61–63].

**Figure 5.**
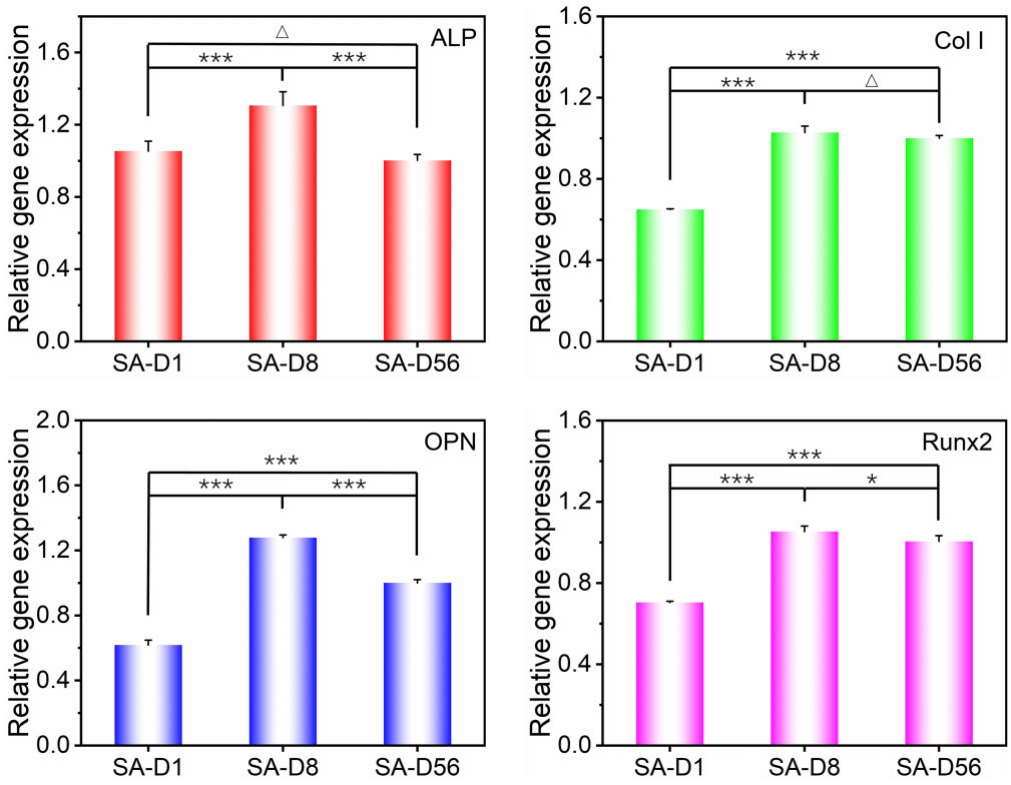
Statistical results of relative osteogenic specific genes (ALP, Col I, OPN, Runx2) expression of bMSCs on the SA fiber patterns with different diameters after 4-day of culture. For each gene, the expression level was normalized to the group of SA-D56. ‘*’: 0.01 < *P *<* *0.05, ‘***’: *P *<* *0.001, ‘Δ’: *P *>* *0.05.

Statistical results of the gene expression are shown in Fig. 5. When compared to the cells on SA-D56, the expression level of ALP, OPN, and Runx2 were upregulated to 1.31-, 1.27- and 1.05-fold, respectively, for the cells on SA-D8, while the Col I, OPN and Runx2 were downregulated to 0.65-, 0.62- and 0.70-fold, respectively, for the cells on SA-D1. In addition, the expression of ALP between the cells on the SA-D1 and SA-D56 and the expression of Col I between the cells on the SA-D8 and SA-D56 showed no significant difference. Overall, the optimal stem cell osteogenesis happened on the fibers with 8 μm in diameter under the sparse arrangement (SA-D8). When the fiber diameter became smaller (SA-D1) or larger (SA-D56), the tendency of cell osteogenesis could decrease.

As for cell differentiation, typical literature has reported that low cell–cell contact, small cell spreading and low cell stress are all unfavorable for the osteogenic differentiation of stem cells and *vice versa* [35, 64, 65]. Moreover, the aspect ratio of cells has also been reported to significantly affect stem cell differentiation. It was found that the optimal osteogenic differentiation happened in the cells with an aspect ratio about 2. At the same time, it would decrease when the aspect ratio of cells became more minor or more significant [52]. Herein, due to the severe restriction of single tiny diameter fibers, cells on the fiber patterns of SA-D1 contracted into a bead-like morphology (petite spreading, see Fig. 3b) and presented a lower aspect ratio (see Fig. 3c). Both are unfavorable for cell osteogenesis, which is probably why the cells on SA-D1 expressed the most defective osteogenic-specific genes. With the increasing fiber diameter, the restriction of cells has decreased and the aspect ratio of cells on the SA-D8 fiber patterns increased to about 2, due to the proper balance of cell restriction and cell contact guidance effects. In addition, cell proliferation was also significantly increased as the fiber diameter increased to 8 μm (see Fig. 4c), thus inducing more cell–cell contact for the cells on SA-D8. The expressions of osteogenic characteristic genes were all significantly up-regulated. As the fiber diameter further increases to about 56 μm (SA-D56), the improved fiber surface area could considerably lead to a lower cell aspect ratio and much lower cell density (less cell–cell contact) when compared to SA-D8. The comprehensive effects of cell–cell communication, cell spreading and cell aspect ratio might result in a lower osteogenic differentiation than SA-D8, but higher than that of SA-D1.

### Effects of fiber diameter on stem cell adhesion and proliferation under dense arrangement

**Figure 6.**
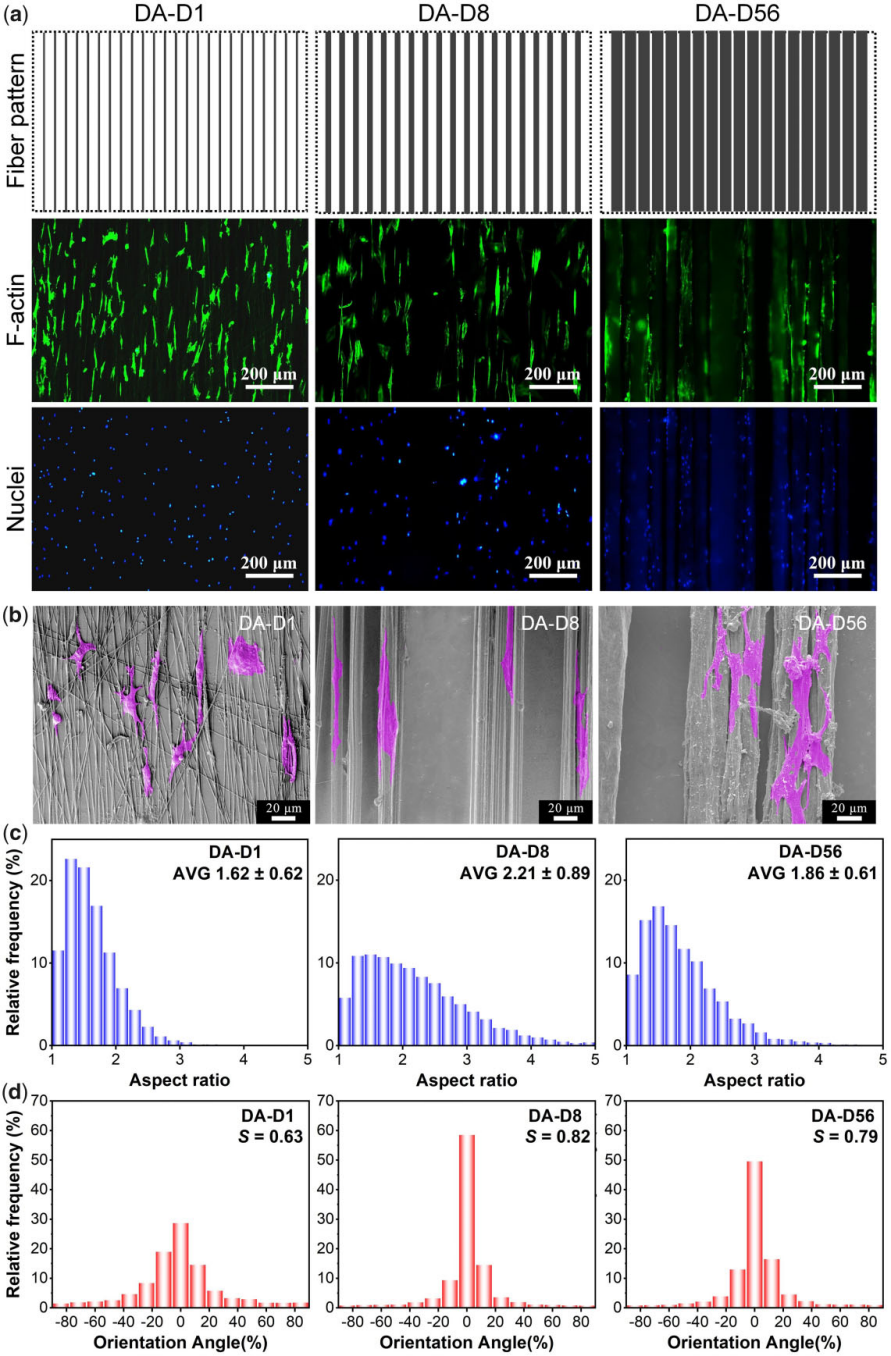
Adhesion of bMSCs on the DA fiber patterns with different fiber diameters after 12 h of culture. (**a**) Fluorescence micrographs of the cells and corresponding cartoon presentation of the fiber patterns. (**b**) SEM micrographs of bMSCs adhesion. (**c**) Statistical results of the aspect ratio distribution of cells. The data of the histograms came from the nuclear aspect ratio of bMSCs. Data on the top-right corner represents mean ± SD of the aspect ratio. (**d**) Statistical results of the orientation angle distribution of cells. The data of the histograms came from the nuclear orientation of bMSCs, and the zero angles were defined along the fiber direction; *S* refers to the order parameter of cell orientation.

Except for the sparse arrangement, we fabricated fiber patterns with a dense structure (see the second row of Fig. 2). We investigated the corresponding fiber diameter effect on stem cell behaviors under this condition, which could be used to mimic traditional fiber platforms with partial ‘interference’ cell adhesion (especially about the cross-adhesion between adjacent fibers) to some extent. After 12 h of culture on the DA fiber patterns with varied diameters, cell cytoskeleton (F-actin) and nuclei were stained. Typical fluorescence micrographs and SEM micrographs are presented in Fig. 6a and b. Results illustrated that cells could quickly form cross-adhesion between adjacent small-diameter fibers on the DA-D1. Evident cross-adhesion between adjacent fibers on the DA-D8 could also be found after 60 h of culture (see Supplementary Fig. S6). Although the distance between adjacent fibers is also very close (smaller than 20 μm) on the DA-D56, the cross-adhesion is hard to be found on DA-D56 within 60 h of culture, probably because the large diameter fibers themselves have sufficient areas for cell adhesion. So, the DA fiber patterns could be used to reveal the fiber diameter combined with composite bonding between adjacent fibers effects on cell behaviors, partially mimicking the condition of traditional fiber mat platforms [41, 43, 44, 66, 67].

Statistical results of the aspect ratio and the orientation angle distribution of cell nuclei are shown in Fig. 6c and d. The average aspect ratios were about 1.62, 2.21 and 1.86 for the cells on the DA-D1, DA-D8 and DA-D56, respectively. In addition, the order parameter (*S*) of cell orientation was about 0.63, 0.82 and 0.79 for the cells on the DA-D1, DA-D8 and DA-D56, respectively.

On the fiber pattern of DA-D1, single fine fibers could seriously restrict cell adhesion. So, the cells prefer extending multiple pseudopods to search for surrounding adhesive fibers, thus quickly forming cross-adhesion between adjacent fibers under a dense arrangement, finally presenting stellate or spindle-shaped cell morphologies (see Fig. 6b). The formed soon cross-adhesions between adjacent fibers in this group gave the nucleus a more circular morphology, resulting in an apparent decrease in aspect ratio (Fig. 6c) and orientation degree (Fig. 6d). In addition, cells on the DA-D8 and DA-D56 were more inclined to adhere along corresponding fibers rather than across adjacent fibers at the initial stage. So, the aspect ratio and orientation degree were significantly higher. Moreover, the cell contact guidance and cell adhesion restriction would gradually weaken with the increasing fiber diameter. So, the aspect ratio and orientation degree of the nucleus were lower on the DA-D56 than on DA-D8.

**Figure 7.**
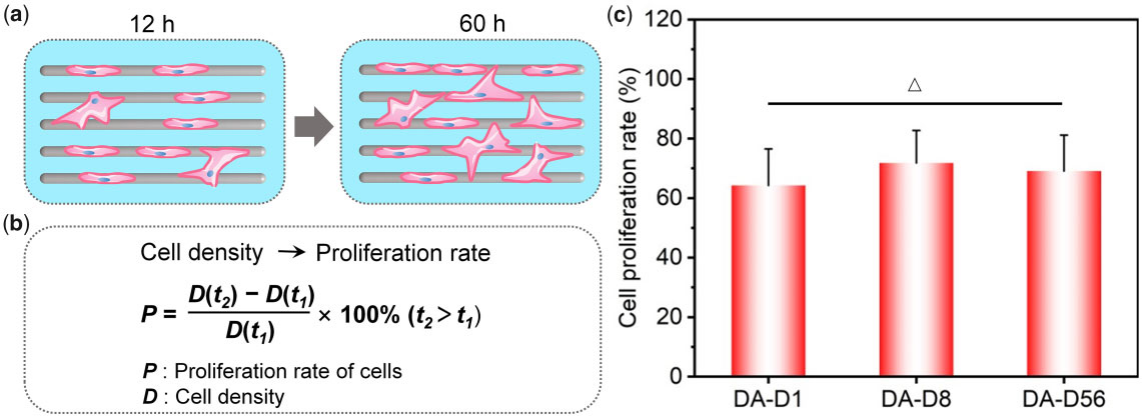
Proliferation of bMSCs on the DA fiber patterns after 60 h of culture. (**a**) Cartoon presentation to illustrate the proliferation of cells. (**b**) Schematic presentation of the calculation of cell proliferation rate from the change of cell density. (**c**) Statistical results of the cell proliferation rate. ‘Δ’: *P *>* *0.05.

After 60 h of culture, the cell proliferation rate was calculated via cell density changing from 12 to 60 h, as schematically shown in Fig. 7a and b. As the fiber diameter increased, the proliferation rates were 64.3 ± 12.2%, 71.8 ± 10.9% and 69.1 ± 12.1%, respectively. The further statistical analysis illustrated that there was no significant difference in cell proliferation rate between each tested group under dense arrangement conditions (see Fig. 7c).

The main reason for this proliferation trend is probably that the DA fibers enabled cells to adhere across the adjacent fibers, providing sufficient ‘adhesive area’ for the cells on each fiber pattern. For the group of DA-D1, cells could quickly form cross-adhesion between adjacent fibers even at the initial stage, thus obtaining the necessary adhesion spot and cell spreading. So, even if the single fiber diameter is as small as 1 μm, cells could still present good proliferation ability. And it is worth noting that the cells on DA-D8 also showed an apparent cross-adhesion between adjacent fibers at 60 h of culture (see Supplementary Fig. S6). The cells gradually perceived significant adhesion limitation of fibers with the increase of cell number, prompting cross-adhesion between adjacent fibers in the dense arrangement condition.

In summary, when cells sensed severe adhesion restriction on the fibers, they stretch out pseudopodia to get more adhesion sites. The DA fibers give them accessible conditions to form cross-adhesion between adjacent fibers. Under dense arrangement conditions, the small diameter fibers restriction on cell adhesion has been significantly weakened by the ‘interference’ cell adhesion between adjacent fibers. That is probably why the cell proliferation rate showed no significant difference between each investigated group (DA-D1, DA-D8 and DA-D56).

### Effects of fiber diameter on stem cell osteogenesis under dense arrangement

**Figure 8.**
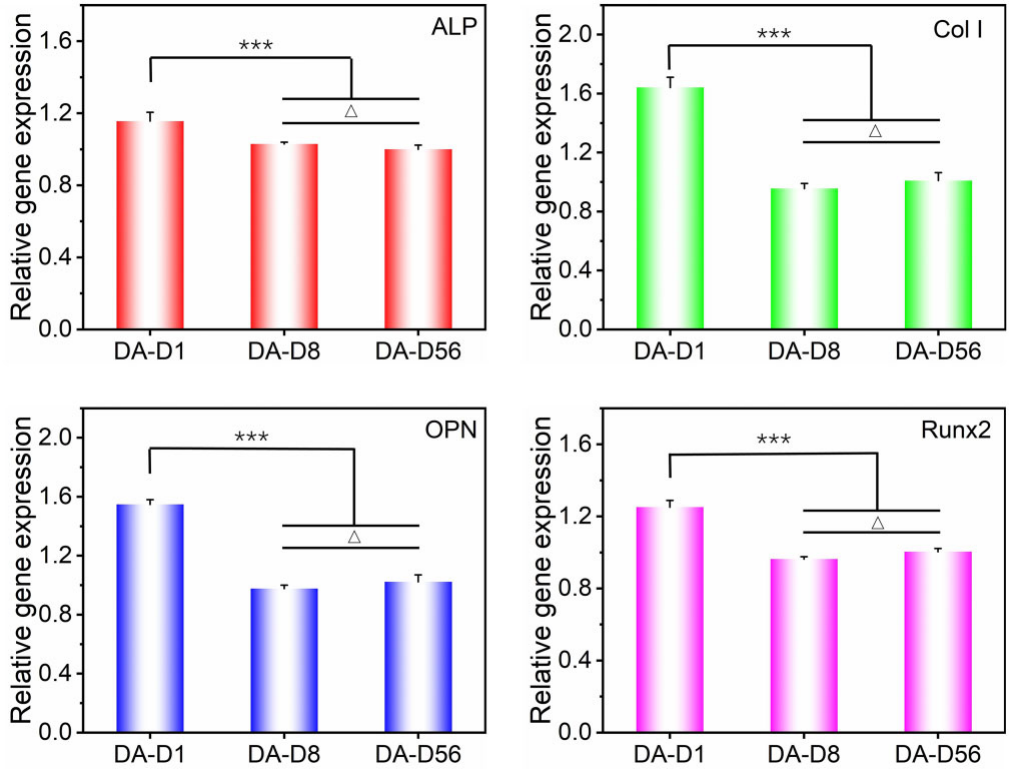
Statistical results of relative osteogenic specific genes (ALP, Col I, OPN, Runx2) expression of bMSCs on the DA fiber patterns with different diameters after 4-day of culture. For each gene, the expression level was normalized to the group of SA-D56. ‘***’: *P *<* *0.001, ‘Δ’: *P *>* *0.05.

Stem cells were cultured on the indicated DA fiber patterns in a growth medium for about 4 days. Then, the expression of ALP, Col I, OPN and Runx2 genes of cells was detected by RT-PCR technology to evaluate corresponding osteogenic abilities. Statistical results of these genes expression are shown in Fig. 8. Compared to cells on the DA-D56, the expression levels of ALP, Col I, OPN and Runx2 were upregulated to 1.16-, 1.64-, 1.55- and 1.25-fold for the cells on DA-D1. The expression of ALP, Col I and OPN was quite similar for the cells between DA-D8 and DA-D56. In addition, the expression of Runx2 was a little higher on DA-D56 than DA-D8. The optimal stem cell osteogenesis happened on the fibers with 1 μm diameter (DA-D1) among the tested groups under the dense arrangement. When the fiber diameter increases to about 8 μm (DA-D8), the tendency of cell osteogenesis could significantly be decreased. When further increasing to about 56 μm (DA-D56), no noticeable osteogenesis tendency changes could be found between DA-D56 and DA-D8.

The enhanced osteogenesis of the cells on DA-D1 was probably due to the following aspects. On the one hand, the majority of the cells could quickly form cross-adhesion between adjacent fibers even at the initial stage of this fiber pattern, which seriously weakened the cell restriction effect of single fine fibers, thus obtained necessary adhesion spot, cell spreading and good cell proliferation (Figs 6 and 7). On the other hand, cell adhesion across multiple fine fibers could lead to large cell protrusions interacting with the delicate fibers and higher cell stress [41, 68], which further promotes stem cell osteogenesis [52, 69]. The combination of these factors significantly improved the osteogenic differentiation of stem cells on DA-D1. On the traditional fiber mats with densely aligned or random fibers, the smaller diameters have also been reported to be more beneficial for cell osteogenesis [43, 44, 70]. As for the cells on the DA-D8 and DA-D56, most of them still adhesion along corresponding fibers. Because the total cell adhesive area (fiber surface) was much more prominent on the DA-D8 and DA-D56 when compared to that of the SA-D8 and SA-D56, the vital cell contact guidance effect on the fibers with 8 μm in diameter and more extendable regions along corresponding fibers make cells on DA-D8 presented an average aspect ratio apparent larger than 2. While relative ‘free’ adhesion on DA-D56 makes cells show an average aspect ratio smaller than 2. In addition, the increased adhesive area also decreases related cell density and cell–cell contact to some extent. These kinds of cell shape changes (deviation from the optimal value of about 2 for osteogenesis [52]) and cell–cell contact cues could decrease the osteogenic differentiation of stem cells on the DA-D8 and DA-D56.

### Comparison of the effects of fiber diameter on stem cell proliferation and differentiation under sparse or dense arrangement

In this study, two types of single-layer and parallel-arranged fiber patterns with varying fiber diameters and excellent non-fouling backgrounds were first designed and fabricated, which could be used to rule out the ‘interference’ cell adhesions in a controllable way. All kinds of ‘interference’ cell adhesions could be effectively excluded on the SA fibre patterns, thus quite suitable for precisely revealing the single fiber diameter cues’ effect on cell behaviors. While the DA fiber patterns could be used to indicate the effects of fiber diameter combined with corresponding cross-adhesion between adjacent fibers on cell behaviors, which could be used to mimic traditional fiber mat platforms with partial ‘interference’ cell adhesion to some extent. Investigating and comparing the stem cell proliferation and osteogenic differentiation of these two types of fiber patterns could be more comprehensive and objective in understanding the influence of fiber diameter cues on stem cell behaviors.

**Figure 9.**
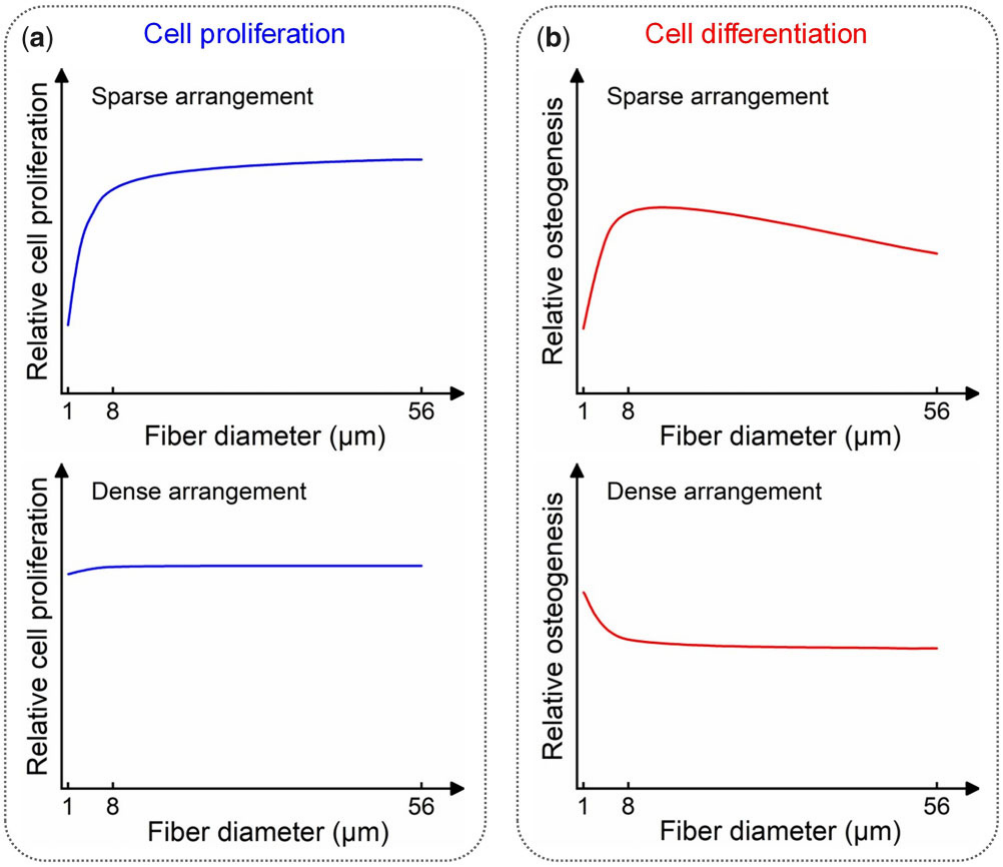
Summary of the bMSCs proliferation and osteogenic differentiation trends on the fiber patterns with varied diameters under sparse and dense arrangements examined in this study. (**a**) Cell proliferation. (**b**) Cell osteogenesis.

The trends of the bMSCs proliferation on the fiber patterns with varying fiber diameters under sparse or dense arrangements examined in this study were schematically illustrated (Fig. 9a). As the fiber diameter increased, the cell proliferation trend was quite different between these two conditions. Under the sparse arrangement, cell proliferation has been seriously inhibited on the tiny diameter fibers (SA-D1). As the fiber diameter increases to 8 μm (SA-D8), cell proliferation has sharply increased. As the fiber diameter further increases from 8 to 56 μm (SA-D56), only a low cell proliferation rate has been increased. Under the dense arrangement, the cell proliferation rate on the tiny diameter fibers (DA-D1) was as high as that on the medium (DA-D8) and large diameter fibers (DA-D56), and there was no significant difference between each group.

The difference in cell adhesion on the same-sized fibers between the sparse and dense arrangement conditions was probably the main reason for these different proliferation trends. Specifically, cells could affect only adhesion on the delicate fibers with 1 μm in diameter on SA-D1, and the restriction of cells was severe, resulting in poor cell adhesion and small cell spreading (contracted into a bead-like morphology, Fig. 3b). So, the cell proliferation on SA-D1 was relatively poor. As the fiber diameter increased, corresponding severe restriction weakened gradually, inducing the cell proliferation rate quickly on SA-D8. Further increase in fiber diameter would not increase cell proliferation, which might be attributed to the comprehensive interaction between cell spreading and cell–cell contact effects on cell proliferation [35, 48]. Under density arrangement, be quite different from SA-D1, cells on DA-D1 could easily cross adjacent fibers to form cross-adhesion even at the initial cell adhesion stage (Fig. 6b), which enables cells to obtain enough space and spots for adhesion, thus seriously weakened the restriction effect of single fine fibers. With the cell number increase, cells on the DA-D8 could also sense corresponding severe restriction and cross the adjacent fibers to form cross-cell adhesion (Supplementary Fig. S6). The cell adhesion between adjacent fibers that happened on the DA fiber patterns could significantly weaken the single small-sized fibers restriction on cell adhesion and proliferation, thus inducing a similar cell proliferation rate between each group during the tested cell culture period.

Osteogenic differentiation of the bMSCs on the fiber patterns with varied diameters under sparse and dense arrangements examined are schematically presented (Fig. 9b). As the fiber diameter increased, the cell osteogenesis trend was also quite different between the sparse and dense arrangement conditions. As for the sparse arrangement, the optimal cell osteogenesis happened on the fibers with about 8 μm diameter (SA-D8). When the fiber diameter decreased to 1 μm (SA-D1), the osteogenic differentiation was sharply reduced, and when the diameter increased to 56 μm (SA-D56), the osteogenic differentiation could be gradually decreased. Under the dense arrangement, it was exciting that the optimal cell osteogenesis happened on the fibers with about 1 μm diameter (DA-D1). As the fiber diameter increased to 8 μm (DA-D8), the osteogenesis gradually decreased. And the osteogenic differentiation has no apparent changes when the fiber diameter further increased from 8 to 56 μm (DA-D56).

Probably due to the severe limitation of cell adhesion, spreading and poor cell stress (contracted into a bead-like morphology, Fig. 3b) caused by the single tiny fibers with 1 μm in diameter, the lowest osteogenesis happened on SA-D1. The slight cell spreading and low cell stress were unfavorable for cell osteogenesis [35, 65]. On SA-D8, the restriction of cells has decreased and the aspect ratio of cells increased to about 2 due to the proper balance of cell restriction and cell contact guidance effects. In addition, cell proliferation was significantly increased on these single fibers compared to SA-D1 (see Fig. 4c), thus inducing more cell–cell contact for the cells on SA-D8. More cell–cell connections and an aspect ratio of about 2 are both reported to be beneficial for cell osteogenesis [35, 52]. As the fiber diameter further increased to 56 μm, the corresponding cell adherent area would be significantly increased and the contact guidance effect would be weakened, thus decreasing the cell–cell contact and cell aspect ratio to some extent. This would gradually reduce corresponding osteogenic differentiation. The optimal osteogenesis happened on the fibers with 8 μm in diameter under the sparse arrangement.

When cells sensed severe adhesion restriction on the single D1 fibers, they stretch out pseudopodia to get more adhesion sites. The dense arrangement of fibers gives them accessible conditions to form cross-adhesions between adjacent fibers. On the one hand, the easily created cross-adhesion between adjacent fibers on DA-D1 could seriously weaken the cell restriction effect of single D1 fibers, thus obtaining necessary cell adhesion, cell spreading and good cell proliferation (Figs 6 and 7). On the other hand, this kind of cell adhesion crosses proper multiple fine fibers, which could lead to large cell protrusions interacting with the fibers and higher cell stress [41, 68]. This further promotes cell osteogenesis [52, 69, 70]. The combination of these factors probably significantly improved the osteogenic differentiation of stem cells on DA-D1. However, the distance between adjacent fibers is also very close (smaller than 20 μm) on the DA-D8 and DA-D56. Most cells still adhere to corresponding fibers because of the large adherent area on the middle and large fibers. The robust cell contact guidance effect on the fibers with 8 μm in diameter makes cells on DA-D8 present an average aspect ratio noticeably larger than 2. While relative ‘free’ adhesion on the large diameter fibers (DA-D56) made the cells show an average aspect ratio significantly smaller than 2. In addition, the increased adhesive area will also decrease corresponding cell density and cell–cell contact. These kinds of cell shape changes (deviation from the optimal value of about 2 for cell osteogenesis [52]) and cell–cell contact cues could probably decrease the osteogenic differentiation of stem cells. So, higher osteogenesis happened on the fibers with 1 μm in diameter under dense arrangement among the tested groups.

The different osteogenesis trends between sparse and dense fiber arrangements have hinted the importance of cross-adhesions between adjacent tiny fibers for enhancing stem cell osteogenesis, thus giving us valuable guidance for designing and modifying corresponding fibrous scaffolds in the bone tissue engineering. In addition, the robust cell contact guidance effect on the fibers with 8 μm in diameter revealed in this literature has also provided essential reference for the design of fibrous scaffolds for repairing tissues with oriented microstructures, such as the tendon and muscle. Overall, based on excluding the ‘interference’ of background adhesion and cross-adhesion between adjacent fibers, the results from SA fiber patterns could precisely reflect the influence of single inherent fiber diameter cues on cell proliferation and osteogenic differentiation. The cell proliferation and differentiation trends on the DA fibre patterns are quite different from those obtained from the SA ones. It was found that the cell adhesion between adjacent fibers under dense arrangement fiber patterns is probably the main reason for inducing these differences. These results could give us a more comprehensive and objective understanding of the effects of fibre diameter cues on the proliferation and osteogenic differentiation of stem cells.

## Conclusions

We designed and constructed two kinds of single-layer and parallel-arranged fiber patterns with typical fiber diameters (1–56 μm) and excellent non-fouling background, named as SA and DA fiber patterns. We comprehensively investigated the fiber diameter effects on stem cell proliferation and osteogenesis based on eliminating the undesired ‘interference’ cell adhesions in a controllable way. The SA fiber patterns indicated that small diameter fiber (SA-D1) could seriously restrict cell proliferation and osteogenesis when compared to the middle (SA-D8) and large (SA-D56) ones, and SA-D8 enhanced cell osteogenesis when compared to SA-D1 and SA-D56. At the same time, cells presented similar proliferation ability and even the highest osteogenic ability on the DA fiber patterns with small diameter fibers (DA-D1) when compared to the middle (DA-D8) and large (DA-D56) ones. In this report, SA fiber patterns could exclude all the ‘interference’ cell adhesions onto the background or adjacent fibers. In contrast, DA fiber patterns could only ban the ‘interference’ cell adhesions onto the background. The cross-adhesions between adjacent fibers under dense fiber arrangement probably are the main reason for inducing these significant differences in cell proliferation and osteogenesis trends along with fiber diameters. Carefully investigating and comparing the results of SA and DA fiber patterns could make scientists understand the effects of fiber diameter on stem cell behaviors more precisely and objectively, thus providing valuable references and guidance for designing and modifying high-efficiency fibrous biomaterials.

## Supplementary Material

rbad001_Supplementary_Data
