# Supplementary material for: Fiber diameters and parallel patterns: proliferation and osteogenesis of stem cells
Source: Regen Biomater. 2023 Jan 12;10:rbad001. doi: 10.1093/rb/rbad001 (PMC9887345; doi:10.1093/rb/rbad001)
Supplement: rbad001_Supplementary_Data [file rbad001_supplementary_data.docx]

Supporting Information

**Fiber diameters and parallel patterns: Proliferation and osteogenesis of stem cells**

Zhanghong Gu^a^, Suna Fan^a^, Subhas C. Kundu^b^, Xiang Yao^a^*, Yaopeng Zhang^a^*

a. State Key Laboratory for Modification of Chemical Fibers and Polymer Materials, Shanghai Engineering Research Center of Nano-Biomaterials and Regenerative Medicine, College of Materials Science and Engineering, Donghua University, Shanghai 201620, People’s Republic of China.

b. I3Bs - Research Institute on Biomaterials, Biodegradables and Biomimetics, Headquarters of the European Institute of Excellence on Tissue Engineering and Regenerative Medicine, University of Minho, Barco, Guimarães, 4805-017, Portugal

*Corresponding authors:

Xiang Yao, Ph.D.

Email: yaoxiang@dhu.edu.cn

Yaopeng Zhang, Ph.D.

Email: zyp@dhu.edu.cn


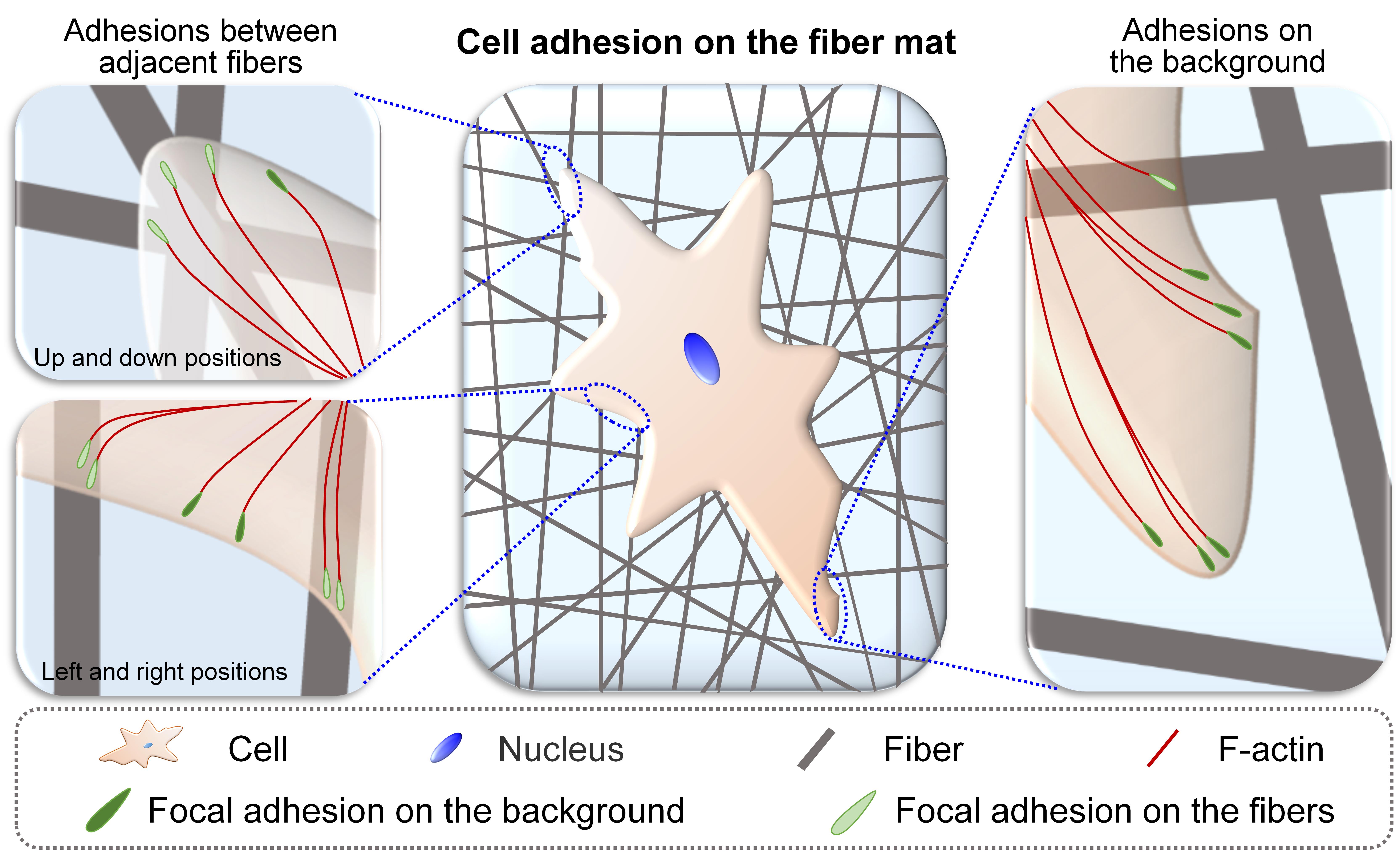


**Figure S1** Animation illustrates the “interference” cell adhesions on fiber mat platform. The “interference” cell adhesions could be mainly divided into adhesions between adjacent fibers and adhesions on the background (such as tissue culture plates).


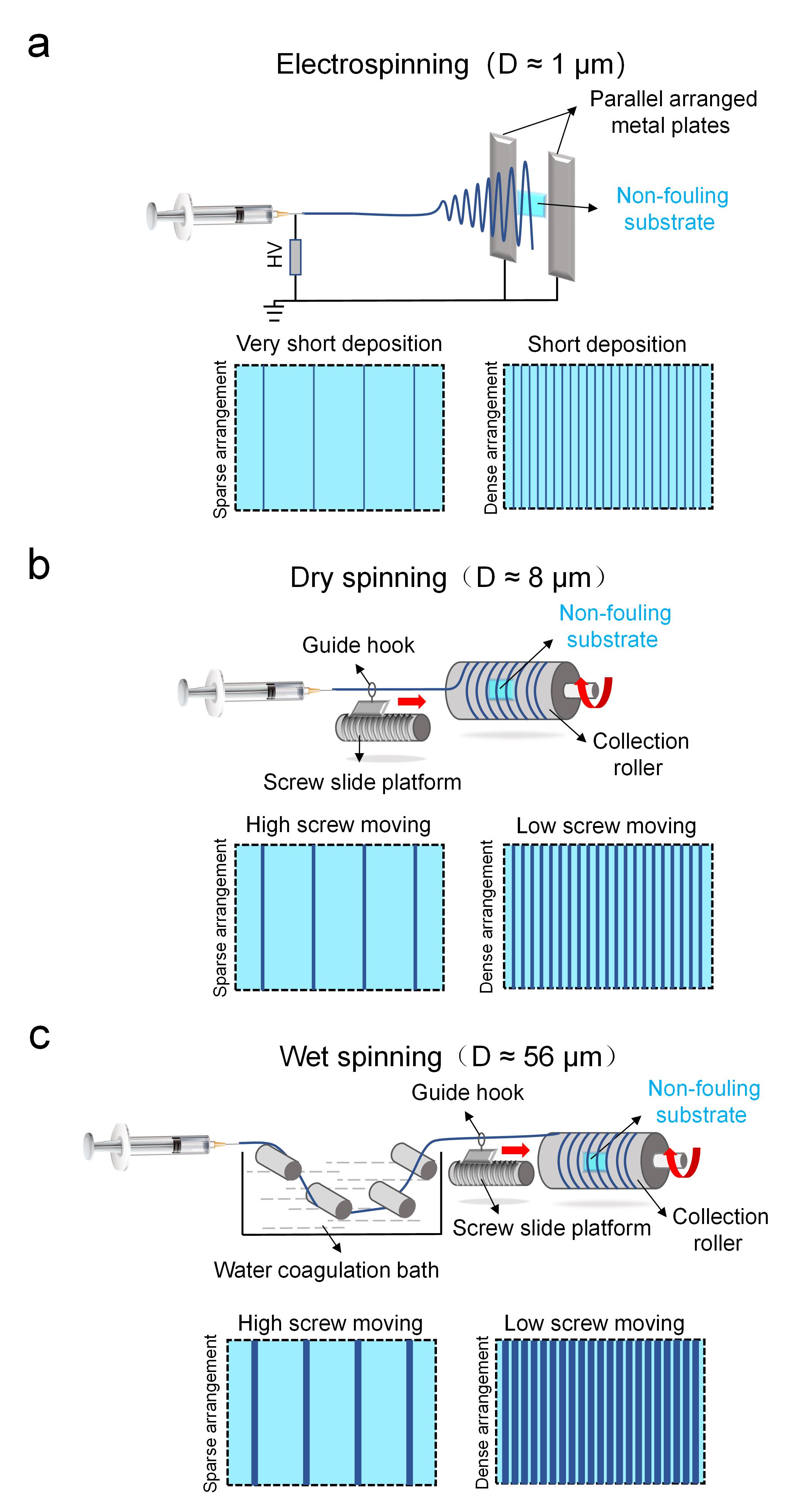


**Figure S2** Schematic illustration of the fabrication of single-layer and parallel-arranged fiber patterns with typical fiber diameters in sparse and dense arrangements. (a) Sparsely and densely arranged D1 fiber patterns fabricated by electrospinning. (b) Sparsely and densely arranged D8 fiber patterns fabricated by dry spinning. (c) Sparsely and densely arranged D58 fiber patterns fabricated by wet spinning.

**Table S1** Statistical results of the fiber diameter and interspacing distance between adjacent fibers in the fabricated single-layer and parallel-arranged fiber patterns.

| Groups | Diameter (μm) | Interspacing distance (μm) |
| --- | --- | --- |
| SA-D1 | 1.2 ± 0.4 | 145.5 ± 35.7 |
| DA-D1 | 1.1 ± 0.2 | 11.9 ± 9.5 |
| SA-D8 | 7.8 ± 2.7 | 161.3 ± 22.9 |
| DA-D8 | 7.6 ± 2.4 | 19.1 ± 12.5 |
| SA-D56 | 55.9 ± 12.3 | 166.0 ± 17.4 |
| DA-D56 | 56.7 ± 13.9 | 8.4 ± 4.5 |


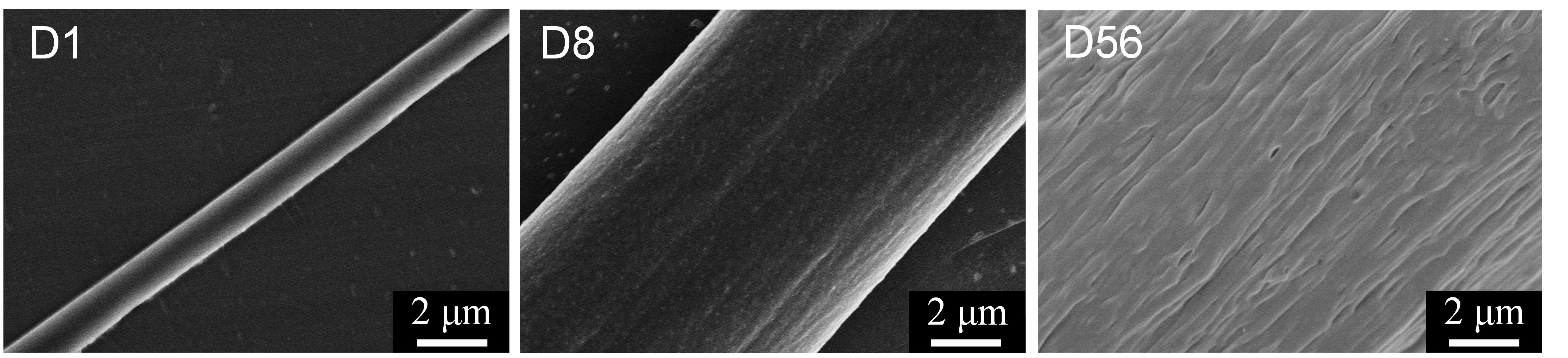


**Figure S3** SEM micrographs of the fiber surface of D1, D8 and D56 fibers.


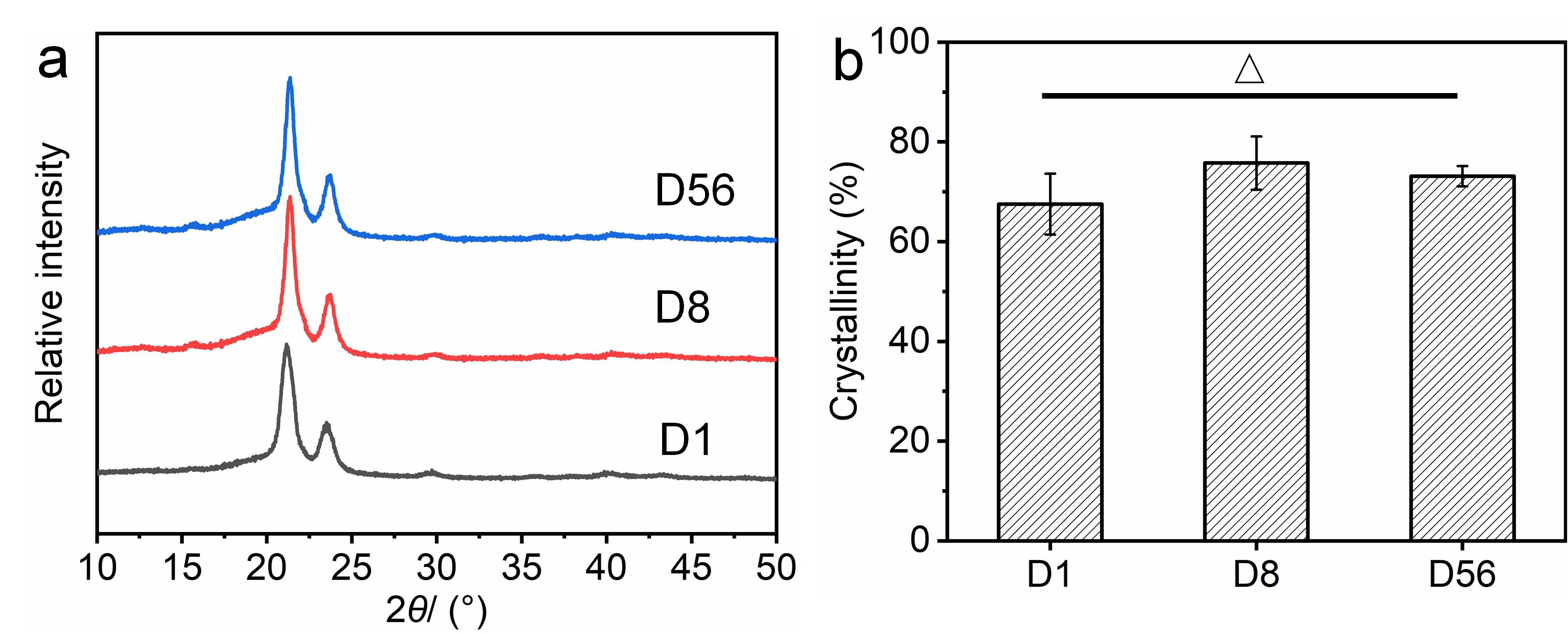


**Figure S4** Crystallinity characterization of the single fibers in the fabricated fiber patterns with different fiber diameters. (a) Typical XRD characterization of corresponding single PCL fibers. (b) The data of XRD calculate the crystallinity of the corresponding single PCL fibers. “Δ”: *p* > 0.05.


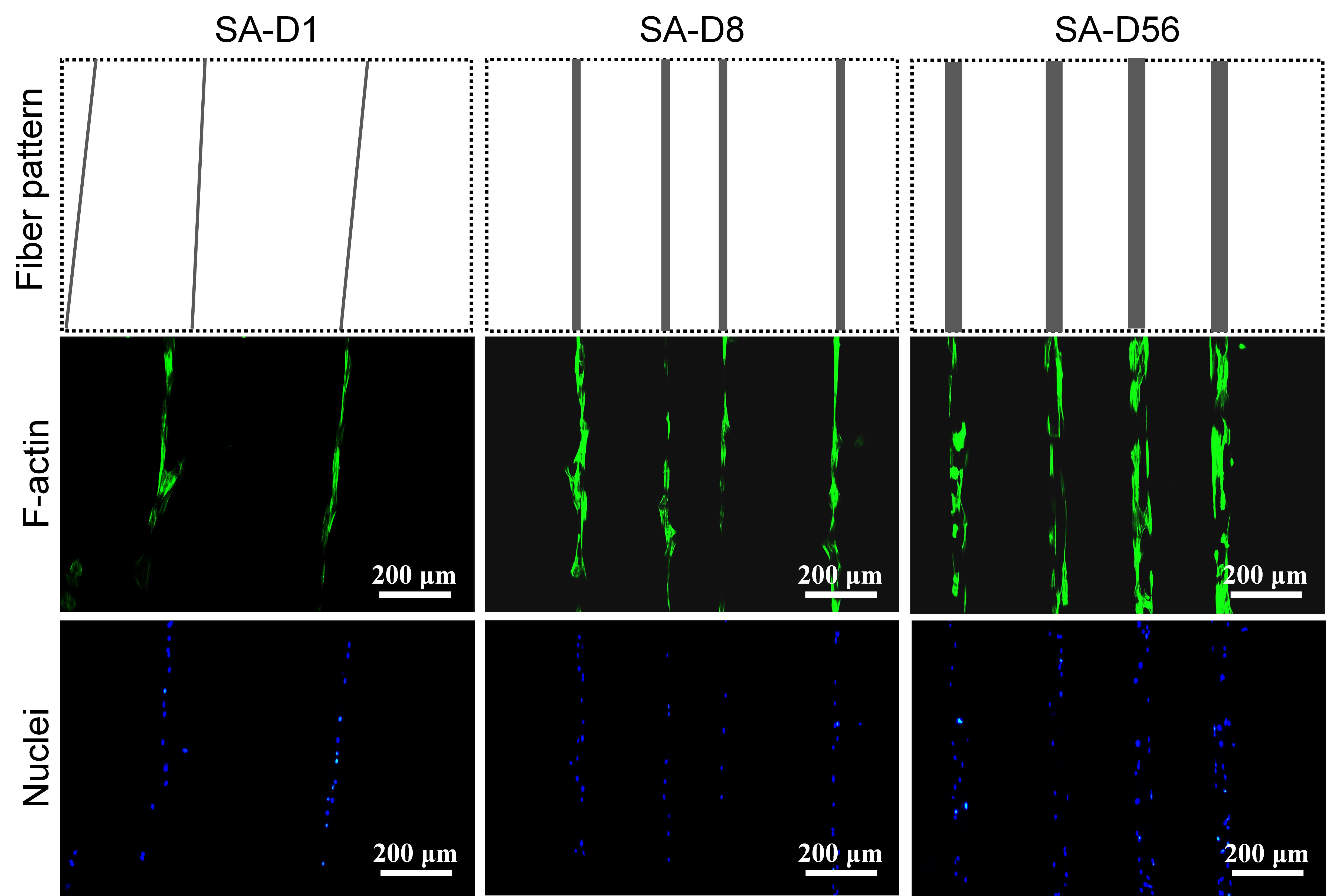


**Figure S5** Fluorescence micrographs of bMSCs adhesion on the sparsely arranged fiber patterns after 60 h of culture and corresponding cartoon presentation of the fiber patterns. Color scheme: Green, F-actins; blue, nuclei.


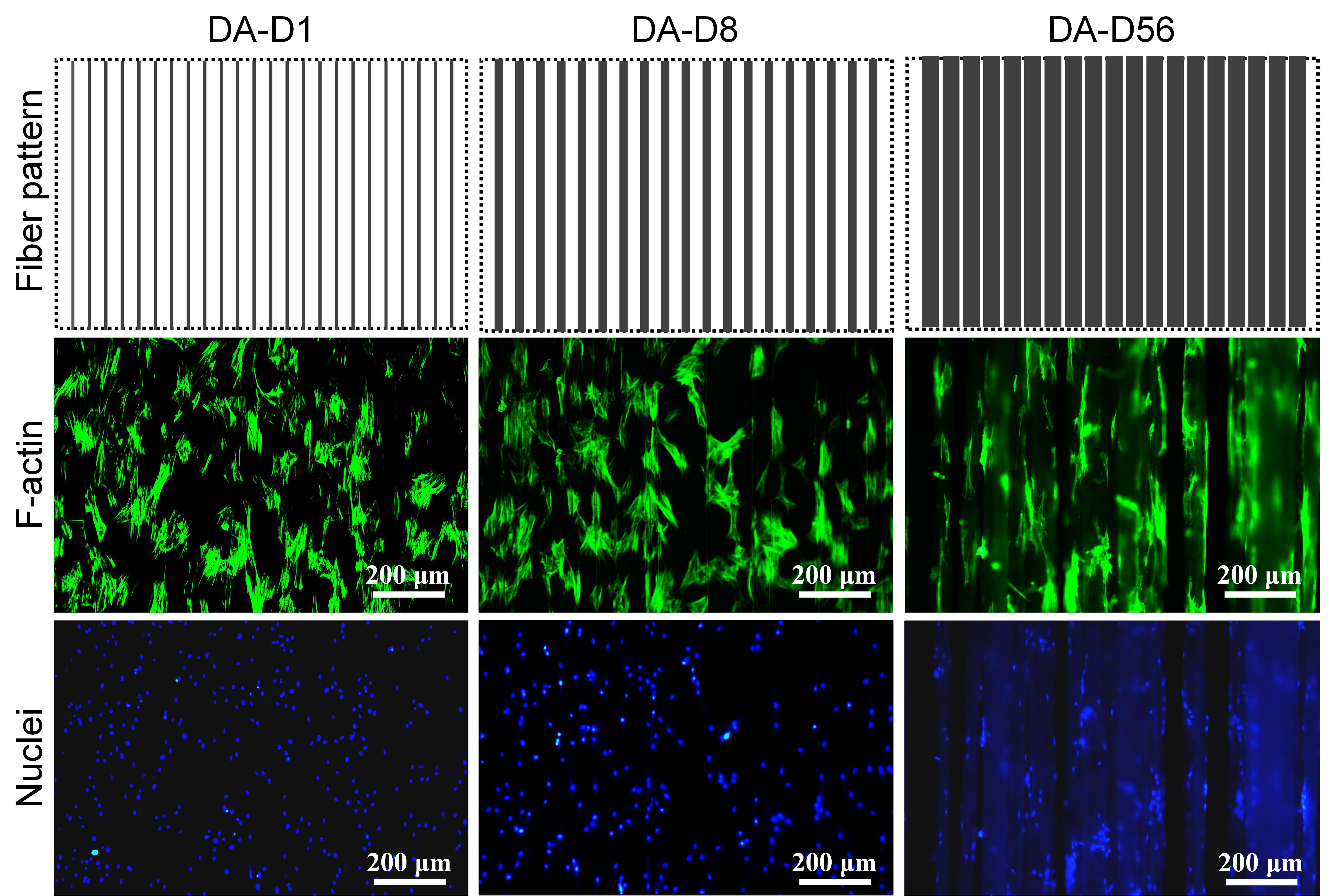


**Figure S6** Fluorescence micrographs of bMSCs adhesion on the densely arranged fiber patterns after 60 h of culture and corresponding cartoon presentation of the fiber patterns. Color scheme: Green, F-actins; blue, nuclei.
